# Fishnition: Developing Models From Cognition Toward Consciousness

**DOI:** 10.3389/fvets.2021.785256

**Published:** 2021-12-15

**Authors:** Paula Droege, Natalie Schwob, Daniel J. Weiss

**Affiliations:** ^1^Department of Philosophy, The Pennsylvania State University, University Park, PA, United States; ^2^Department of Psychology, The Pennsylvania State University, University Park, PA, United States; ^3^Department of Psychology and Program in Linguistics, The Pennsylvania State University, University Park, PA, United States

**Keywords:** animal consciousness, flexibility, Temporal Representation Theory, dual-systems theory, trace conditioning, unlimited associative learning (UAL)

## Abstract

A challenge to developing a model for testing animal consciousness is the pull of opposite intuitions. On one extreme, the anthropocentric view holds that consciousness is a highly sophisticated capacity involving self-reflection and conceptual categorization that is almost certainly exclusive to humans. At the opposite extreme, an anthropomorphic view attributes consciousness broadly to any behavior that involves sensory responsiveness. Yet human experience and observation of diverse species suggest that the most plausible case is that consciousness functions between these poles. In exploring the middle ground, we discuss the pros and cons of “high level” approaches such as the dual systems approach. According to this model, System 1 can be thought of as unconscious; processing is fast, automatic, associative, heuristic, parallel, contextual, and likely to be conserved across species. Consciousness is associated with System 2 processing that is slow, effortful, rule-based, serial, abstract, and exclusively human. An advantage of this model is the clear contrast between heuristic and decision-based responses, but it fails to include contextual decision-making in novel conditions which falls in between these two categories. We also review a “low level” model involving trace conditioning, which is a trained response to the first of two paired stimuli separated by an interval. This model highlights the role of consciousness in maintaining a stimulus representation over a temporal span, though it overlooks the importance of attention in subserving and also disrupting trace conditioning in humans. Through a critical analysis of these two extremes, we will develop the case for flexible behavioral response to the stimulus environment as the best model for demonstrating animal consciousness. We discuss a methodology for gauging flexibility across a wide variety of species and offer a case study in spatial navigation to illustrate our proposal. Flexibility serves the evolutionary function of enabling the complex evaluation of changing conditions, where motivation is the basis for goal valuation, and attention selects task-relevant stimuli to aid decision-making processes. We situate this evolutionary function within the Temporal Representation Theory of consciousness, which proposes that consciousness represents the present moment in order to facilitate flexible action.

## Introduction

A challenge to developing a model for testing animal consciousness is the pull of opposite intuitions. On one extreme, the anthropocentric view holds that consciousness is a highly sophisticated capacity involving self-reflection and conceptual categorization that is almost certainly exclusive to humans. At the opposite extreme, an anthropomorphic view attributes consciousness broadly to any behavior that involves sensory responsiveness. Yet human experience and observation of diverse species suggest that the most plausible case is that consciousness functions between these poles (1, 2). Subjectively, everyday conscious activity seems to occur without reflective thought, and a great deal of behavior (habits, conditioned response, reflex) seems to occur without consciousness. Objectively, single-celled animals respond to chemicals in their environment but display no other characteristics indicative of consciousness, whereas the behavior of mammals does seem to indicate consciousness despite the likely absence of self-reflection.

One source of opposing intuitions is the lack of agreement on how to define consciousness. Consensus is forming around the idea of *phenomenal consciousness* as the appropriate target for explanation (3–5). However, the definition of phenomenal consciousness is problematically vague: “what it's like” to have a sensation or thought, its feeling or qualitative character. Anthropocentric approaches emphasize the subjective awareness of conscious experiences while anthropomorphic approaches emphasize the quality of feeling. We propose that an evaluation of the pros and cons associated with a “high level” anthropocentric approach and a “low level” anthropomorphic account will help identify central features of consciousness.

The dual systems model is a “high level” approach. According to this model, *System 1* can be thought of as unconscious; processing is fast, automatic, associative, heuristic, parallel, contextual, and likely to be conserved across species. Consciousness is associated with *System 2* processing that is slow, effortful, rule-based, serial, abstract, and exclusively human (6, 7). An advantage of this model is the clear contrast between heuristic and deliberation-based responses, but rational deliberation is a very sophisticated cognitive ability that is difficult to demonstrate even in cognitively advanced species such as primates. We also review an example of a “low level” anthropomorphic model involving trace conditioning, which is a trained response to the first of two paired stimuli separated by an interval (8). This account highlights the role of consciousness in maintaining a stimulus representation over a temporal span, though it overlooks the importance of attention in subserving and also disrupting trace conditioning in humans (9).

Through a critical analysis of these two extremes, we develop the case for flexible behavioral response to the stimulus environment as the best model for demonstrating animal consciousness. Flexibility can be defined as the ability to adapt both goals and actions to situational demands. We discuss a methodology for gauging flexibility across a wide variety of species and offer a case study in spatial navigation to illustrate our proposal. Flexibility serves the evolutionary function of enabling the complex evaluation of changing conditions, where emotions establish the motivational basis for goal valuation, and attention selects task-relevant stimuli to aid decision-making processes (10–12).

We situate this evolutionary function within the *Temporal Representation Theory* of consciousness, which proposes a definition of consciousness as a representation of the present moment. “What it is like” to have conscious experience is to represent things (feelings, thoughts, events) as happening now. Critically, representation of the present moment is necessary for flexible action (13).

This article originated in a research group on emotion and consciousness led by Victoria Braithwaite, and she contributed significantly to the development of the approach we propose. Victoria was convinced that emotion contributed the differential valuation to environmental conditions that is essential to consciousness. This conclusion followed her ground-breaking work on fish pain, concisely laid out in her book (14) and subsequent work on emotion and consciousness (12, 15).

For Victoria, the question of whether fish can feel pain was a moral concern. Dismantling a laptop or a robotic vacuum cleaner is not an ethical problem, because these machines do not feel pain. In contrast, we have laws to protect dogs, cats, and livestock, because we have good reason to believe that mammals are conscious and therefore suffer when injured. If fish are also conscious, regulations should be developed to ensure suffering of these animals is limited as well (16).

## Model 1: Deliberation Beyond Heuristics

If “high level” approaches are correct, however, then fish are most definitely not conscious. On this sort of model, self-reflective deliberation or other sophisticated cognitive ability is necessary for consciousness. Several contemporary theories posit the sense of self as central, based on the way introspection reveals human experience [for a review see (17)]. When we attend to conscious thoughts and sensations, we always find a self as the subject of those experiences. Accordingly, philosophers and psychologists have long discussed two modes for reasoning. One is fast, automatic, associative, and implicit, while the other is slower, effortful, deliberate, and explicit [(6, 7); see (18) for review]. These modes have come to be known as System 1 and System 2 respectively (19) and have recently risen to prominence in their application toward decision-making, as many in the field of behavioral economics have embraced dual system constructs [e.g., (20)].

Given that decision-making is a fundamental survival process for all organisms that are candidates for consciousness, it is natural to wonder whether the dual systems model might lend insight into adjudicating conscious from non-conscious species. Correspondingly, System 1 has sometimes been assumed to share a lengthy evolutionary history with other species, while System 2 is typically cast as uniquely human [e.g., (18)]. However, there are real ambiguities between how System 1 and System 2 map on to the distinction between non-conscious and conscious processes (21).

In his book, *Thinking Fast and Slow*, Kahneman (20) describes a dichotomy between the Experiencing Self (System 2), and the Remembering Self (System 1). The contrast is based on a series of studies in which subjects endured unpleasant events or procedures and subsequently were asked to rate their experience or their willingness to reengage in a similar experience (22). The results were surprising; subject ratings of unpleasantness were not correlated with the overall amount of discomfort endured. Rather, evaluation of the experience was primarily influenced by the peak intensity of pain as well as the amount of pain at the end of the procedure (known as the peak-end effect), while neglecting the overall amount of time the event or procedure lasted (duration neglect). For example, using a procedure known as the cold pressor task, Kahneman et al. (22) immersed subjects' hands in very cold water. In the first condition, the temperature remained constant (14°C) for 60 s, while in the second condition, there was an identical 60 s immersion followed by an additional 30 s of immersion in which the water was one degree warmer (a perceptible difference). The majority of subjects reported a preference for repeating the second condition over the first, despite the fact that it entailed a prolonged period of discomfort. However, participants did prefer the shorter trial over the long one if the experimenters described the two conditions (22, 23). This suggests a dichotomy between the fast, automatic judgment (conforming to the peak-end heuristic) and the slow, deliberative judgment. As a model of consciousness, it is tempting to attribute fast, heuristic responses to unconscious processing, while slow, deliberative responses demand consciousness.

Applying this model to animals, the first question is whether the peak-end effect is evolutionarily ancient. This can be addressed using the comparative method and looking for homologous behavioral choices made by closely related species. While initial investigations suggested that peak-end effects were not shared with monkeys (24), later studies found that, like humans, rhesus monkeys do pay disproportionate attention to the peak and endpoint of an event (25, 26). For instance, rhesus monkeys preferentially choose sequences of rewards in which the highest values are located toward the end rather than middle of the sequence. These studies suggest that the peak-end rule likely shares a lengthy evolutionary history (although to the best of our knowledge, these studies have not extended beyond primates).

Given the strong possibility that peak-end effects are evolutionarily conserved, it is natural to wonder whether other species might exhibit a different set of preferences were they able to engage in deliberation, much like human subjects in the cold-pressor experiment described above. Here too, there is preliminary evidence that, like humans, non-humans express preferences that do not abide by peak-end effects when the problem is framed in a way that encourages deliberation. In a comparative study, Egan Brad et al. (26) found that humans and capuchin monkeys exhibited peak-end preferences when choosing reward sequences. However, in a follow-up experiment in which subjects were required to create their own reward sequences, neither humans (both adults and children were included) nor capuchin monkeys created sequences that accord with peak-end rules. Ultimately, there are likely too many differences between the choice task and the sequence-assembly task to infer that the latter involved rational deliberation [see (26, 27)].

According to the dual systems approach to consciousness, decisive evidence of rational deliberation would count as evidence of consciousness. The primate research falls just short of that bar in showing that capuchin monkeys are not subject to peak-end rules in a prospective reward task. These findings underscore the important difference between following a heuristic and making a deliberate choice. We would expect that animals capable of consciousness should not be “stuck” conforming to responses dictated by evolutionarily ancient heuristics [see (28)]. Rather, they should be able to overcome these rules in favor of choices that reflect anticipated preferences. In humans, deliberation of this kind always requires consciousness, so evidence for deliberate decision-making is reasonably taken as evidence for consciousness.

However, evidence of deliberative decision-making is a very high bar. Not only is it difficult to conduct appropriate experiments, as with the peak-end research, but there is a tendency to interpret all animal behavior as merely associative. Morgan's Canon advises that explanations of behavior involving simple mechanisms are preferable to explanations involving more complex cognitive abilities. While there is merit to this approach, it should not be used in every case (29). For example, evidence suggesting episodic-like memory in scrub-jays has been criticized as insufficient due to the possibility of accounting for the behavior in terms of associative learning (30, 31). Whether or not the ascription of episodic memory is appropriate, scrub-jays clearly demonstrate cognitive capacities beyond simple association (32).

The challenges of demonstrating homologs of System 2 processing in animals, coupled with the widely held view that mammals, birds, and possibly other animals are conscious (16, 33), provides a reason to look for a more tractable method. Later in the essay we will consider a case study for demonstrating flexible behavior that deviates from simple association yet bears none of the hallmarks of deliberation (e.g., slow, rule-based, abstract). Whereas it is unlikely that many non-human animals are capable of human-like System 2 processing, flexibility may be widespread in the animal kingdom.

## Model 2: Trace Conditioning Beyond Simple Association

In contrast to high-level approaches that adopt an anthropocentric self-reflective view of consciousness, low-level approaches take an anthropomorphic stance, where any form of sensory responsiveness is interpreted as conscious. For example, trace conditioning is a trained response to the first of two paired stimuli separated by a temporal interval. In their well-known study, Clark and Squire (8) presented a neutral conditioned stimulus (CS) such as a tone to participants, followed after a short interval by a motivationally significant unconditioned stimulus (UCS) such as an air puff. Because participants acquired a conditioned response to the CS only when they reported awareness of the tone-air puff contingency, Clark and Squire concluded that conscious knowledge of the CS-UCS relation is necessary for trace conditioning. A number of researchers have used this conclusion to argue that animals with neurologically simple systems such as honeybees and drosophila are conscious, because they too successfully learn by trace conditioning (34, 35).

While consciousness may be much more widespread in the animal kingdom than previously thought, it is counterintuitive to think that learning a very basic association is sufficient to demonstrate consciousness. Evidence that trace conditioning is successful even when the CS was masked and also when patients are in a vegetative state (36) further casts doubt on the link between consciousness and trace conditioning. Moreover, interpretations of trace conditioning experiments are various and subject to critique (37, 38), making it difficult to assess available evidence without a better sense of exactly what is involved in successful and unsuccessful trace conditioning.

Some insight into the source of the disagreements can be gained by examination of the important role played by attention in preventing *both* trace conditioning and awareness of the CS-UCS contingency. In the Clark and Squire (8) study, participants were instructed to watch a silent movie while the trace conditioning stimuli were presented, and there is good reason to think the distraction accounts for the failure of conditioning. What merits investigation is the way that such a basic association can be blocked by attentional inhibition.

Elsewhere (9) we hypothesize that only animals capable of task-directed attention will fail to trace condition under distraction when they are otherwise able to learn this sort of contingency. By filtering task-relevant stimuli, attention serves a critical role in maintaining focus. Consciousness has been linked with the selection and integration of stimuli in the performance of novel, context-dependent tasks, such as watching a movie (39, 40). Thus, both attention and consciousness are necessary to focus on a task, though the two processes are distinct (41, 42).

Trace conditioning without distraction forms a straightforward link between stimuli, albeit over a brief temporal interval. Simple coincidence detectors can account for the formation of these associations without the additional neural resources provided by consciousness or attention. Trace conditioning in insects likely involves strengthening synaptic connections either through prolonging the CS trace or anticipating US activation. Possible mechanisms include recurrent firing of CS-UCS pairing or neuromodulators to maintain the CS over the temporal interval (34). In contrast, trace conditioning in vertebrates requires more complex structures, such as the hippocampus and cerebellum (43).

We suggest that these more complex memory structures work in tandem with attentional selection. The ability to focus on one task over alternative possible tasks accounts for the difference in the Clark and Squire experiments between participants who successfully conditioned to the CS and those who failed to condition. Participants who successfully blocked the trace conditioning stimuli in order to focus on the movie failed to report those stimuli and failed to condition to the association. Participants who noticed the trace conditioning stimuli reported and conditioned to them. Attention to the stimuli was coupled with consciousness of the stimuli, as indicated by subjective report, and resulted in conditioning to the CS-UCS relation. The interesting result in the Clark and Squire experiments, on our analysis, is the *failure* to trace condition by participants who almost certainly would have successfully learned the association in the absence of distraction. Consequently, successful trace conditioning alone is insufficient as a test for consciousness [see (9) for further details]. Rather, we argue that having the flexibility to trace condition or not depending on attentional capacity might prove a better index, as we elaborate further in the final model under consideration.

## Model 3: Behavioral Flexibility

The critique of anthropocentric high-level approaches on one extreme and anthropomorphic low-level approaches on the other extreme forms the basis of an emerging realization of the difficulties in establishing a methodology for investigating animal consciousness (1, 2). Theories of human consciousness cannot be extended to animals without running the risk of applying them too narrowly (high-level approaches) or too broadly (low-level approaches). Shevlin (2) calls this the specificity problem and argues that markers of consciousness (clusters of properties associated with conscious processes) can sort between likely and unlikely candidates for consciousness. Markers pick out candidate species for comparative analysis. According to Shevlin, behavioral and physiological similarities across candidate species help establish the correct level of specificity for application of a theory in order to minimize false-negatives and false-positives. For example, a neuroscientific theory based on human consciousness could use a set of markers to determine homologous neural structures in candidate species. Rather than say that fish do not feel pain, because they do not have a cortex (38), the presence in fish of a marker for consciousness such as multi-modal sensory integration (44) would support the suggestion that the telencephalon serves a similar integrative function as cortical structures (45, 46).

We are in favor of Shevlin's “dynamic equilibrium” between theory and a more open-ended cluster approach, with one crucial addition: the functional consequences of consciousness should be a guiding constraint. Evolutionary considerations favor the assumption that consciousness serves a selective advantage. Our suggestion is that flexibility best satisfies the functional, behavioral, and physiological considerations relevant for testing animal consciousness. Flexible behavior, that is, the ability to adapt both goals and actions to situational demands, is connected with the value of attentional selection and inhibition in accomplishing complex, novel tasks. In overriding peak-end effects, rational deliberation demonstrates the power of sustained attention to the individual elements of an experience rather than relying on a faulty memory of the events. Likewise, sustained attention to the movie disrupts trace conditioning. The challenge is to clarify exactly what flexibility involves, and how it charts a middle course between high-level reflective deliberation and low-level associative conditioning.

The first step in meeting this challenge is to identify the sort of cognitive and environmental factors involved in performing the proposed function (physiology is important as well but will not be included here for the sake of brevity). Droege and Braithwaite (12) offered four ways to demonstrate behavioral flexibility using fish as a model species. (1) Differential response to the environment involves the ability to alter behavior to a situation depending on a momentary means-ends assessment. As the most general description of flexibility, this category subsumes the three other forms of demonstration. We list them separately to highlight various ways flexible behavior appears in animals, not to suggest they are mutually exclusive. While all organisms capable of learning utilize past stimulus-response associations to determine action, flexible animals are capable of both goal-selection and action-selection. For example, the cleaner wrasse *Labroides dimidiatus* feeds on the parasites of client fish in a delicate relationship that requires evaluating multiple features of each interaction. Options include biting the flesh of the client fish instead of the parasites or providing a fin massage to soothe the client. Relevant factors in the assessment of options involve past interactions with the individual and the potential response of other clients that are observing the interaction (47–49). In other words, a cleaner wrasse demonstrates behavioral flexibility in its ability to tailor its goals and its actions to ongoing changes among multiple situational factors.

(2) Appropriate response in a novel situation requires the use of past information in a new way. This sort of behavior goes beyond simple conditioning, because it involves combining learned associations and applying them in a stimulus environment that has never been encountered. In a remarkable experiment, male cichlids observed conspecifics of similar size with variable fighting strength from A (strongest) to E (weakest). After watching A beat B, B beat C, C beat D, and D beat E, the observer fish was forced to choose whether to fight B or D. The cichlid appropriately combined the information about fighting strength to choose the marginally weaker opponent, D (50). Because the fish had not seen B fight D, its response to this novel situation, guided by inferential reasoning, demonstrates flexibility.

(3) Manipulation of the environment to accomplish goals exemplifies the ability to refrain from directly acting on a goal in order to better achieve it through indirect action. Tool use is one way to bring about a result by focusing first on something else. Facing the problem of a food pellet too large to eat, a six bar wrasse *Thalassoma hardwicke* carried the food to a pre-selected rock in order to smash the pellet into smaller pieces (51). Though this behavior fails strict criteria for tool use that rule out using substrate as a tool (52), the six-bar wrasse shows that it can refrain from acting on its goal of eating the pellet and pursue an alternate strategy as an intermediate step. Even if the discovery of the pellet-smashing rock was a matter of chance or trial and error, the use of the rock a second time required the ability to remember it and recognize its value as a tool in the new situation.

(4) A final way to demonstrate flexibility may seem too high level: the explicit representation of absent objects. However, this ability does not require that animals understand either the concept of an absent object or of an explicit representation. In fact, something as straightforward as the ability to represent an unrealized goal fulfills this condition (53). What makes the representation explicit is that it is not simply the end of a chain of behaviors; the goal is represented independently. For example, the small goby fish *Bathygobius soporator* needs to represent the terrain of pools surrounding its home pool in order to safely escape predators during low tide (54, 55). Though the adjacent pools cannot be seen, the goby can use a spatial representation learned at high tide to jump from one pool to the next. This sort of navigation demonstrates a more complex representation of spatial relationships than the ability to use a landmark or a series of spatial cues to achieve a goal (56).

An important justification for a behavioral flexibility approach to animal consciousness is the ability to situate functional, physiological, and ecological indicators within an evolutionary context. Our proposal is consonant with an extensive evolutionary argument by Ginsburg and Jablonka (57) that conceives of consciousness as an evolutionary stage rather than a property or process, a form of life rather than an acquired trait [see also (58)]. Conscious animals have “temporally persistent, dynamic, integrated, and embodied neurophysiological states that ascribe values to complex stimuli emanating from the external world, from the body, and from bodily actions” [(57), p. 7]. Their reason for this description of consciousness mirrors our own: consciousness evolved so that animals could respond flexibly to changing environmental and internal conditions. Emotions are critical to this evolutionary process, because they are means by which stimuli are evaluated. Anger, fear, and other negative emotions signal avoidance of stimuli, whereas joy, excitement, and other positive emotions signal approach to stimuli. Though emotional evaluation arguably can occur unconsciously (59), the capacity for assessing complex stimuli is necessary for flexible response.

The evolutionary frame provided by Ginsburg and Jablonka locates consciousness in the middle level of evolutionary development, where each of three levels is structured by a goal that determines its features. At the most basic level, the goal of life is survival and reproduction. The goal of the next level, consciousness, is value-based, action-guided learning. At the most cognitively advanced level, the goal of rationality is normative standards for cultural cooperation. Transitions from one level to the next involve the acquisition of necessary features that “accumulate, combine, and then become sufficient” to constitute the new level [(57), Ch 1]. Their evolutionary approach usefully articulates the mechanisms and dynamics that drive transitions from one level to the next. Gray areas between transitions can be better understood in terms of which mechanisms are operating (or not) and how they interact (or not) [(57), p. 10–17].

On this view, a transition marker is a key feature that indicates achievement of each level of development. Evidence for a transition marker demonstrates that the required coevolved set of mechanisms is in place. In contrast to a criterion, absence of a transition marker does not mean the absence of the system that enables a particular level of development. One or another of the mechanisms may be malfunctioning even though the system is in place. In locked-in syndrome, for example, complete loss of muscle control makes flexible sensorimotor behavior impossible with the exception of eye movements and blinking. Nonetheless, people with locked-in syndrome do not lose their capacity for consciousness (60). The malfunction of one component of the system, even a very important component such as muscle control, does not necessarily eliminate consciousness. This systems approach to analyzing evolutionary development is more coherent and specific than the tendency to produce lists of characteristics associated with messy concepts like life and consciousness. Yet it is less rigid than a set of necessary conditions.

For example, Ginsburg and Jablonka propose unlimited associative learning (UAL) as the transition marker that indicates consciousness. UAL is the “ability to attach motivational value to a compound, multifeatured stimulus and a new action pattern and to use it as the basis for future learning” [(57), p. 3]. Consciousness results from a system that enables UAL, so wherever UAL appears, consciousness appears as well. However, the absence of UAL in evolved animals does not imply lack of consciousness, since the enabling system may be malfunctioning in one way or another.

Generalizing from Ginsburg and Jablonka, we propose that an enabling system for flexibility includes the following: neuron structures to support learning; the development of neural patterns to integrate multi-modal stimuli in a novel situation and respond with complex action sequences; an emotional valence system to differentially weight the value of stimuli, actions, and goals according to a common currency; and an attentional system to select task-relevant stimuli for further processing and inhibit irrelevant stimuli [for more support of these features, see (12, 57, 58)]. Indicators for these elements of the enabling system could be tested in a variety of behavioral and physiological ways, and this evidence would add support to the tests for flexibility described earlier.

One essential piece missing from the evolutionary story offered by Ginsburg and Jablonka is a convincing reason to identify UAL with consciousness. They list hallmarks of consciousness, such as global availability, selective attention, and stimulus integration, and show how UAL depends on the structures that underwrite these hallmarks. Still, there remains the question of why global availability, selective attention and so forth must be conscious. That is, the explanatory gap remains between functions the brain performs and the subjective experience that correlates with it. In the final section, we will construct a bridge across the explanatory gap: a description of consciousness in terms of its function. Before we get to the abstract connection between consciousness and function, the next section will discuss a concrete study to test flexibility.

## A Case Study for Testing Flexibility

In this section, we are proposing tests of navigation as a fruitful test of animal flexibility. One domain where flexibility may be particularly impactful is spatial navigation. Individuals must appropriately shift behavior if something in their landscape changes. Historically, spatial navigation research has been prioritized in mammals (61, 62), though fish have been shown to have remarkable spatial knowledge as well. Salmon are able to return to their birthplace to spawn using olfactory cues (63), and when faced with a novel maze, goldfish (*Carassius auratus*) are able to utilize allocentric cues outside of the tank to find a food compartment (46). These types of navigation, where an individual applies previously acquired information, demonstrate variability but not flexibility in the way we have defined it. The goldfish viewed the environment when completing the initial maze, encoded the allocentric cues, and then utilized these cues when completing a different maze. The fish were not required to transform the information in any way, nor decipher between multiple correct options. Flexibility can be seen in navigation by changing a past “correct” behavior in favor of a better solution.

**Figure 1:**
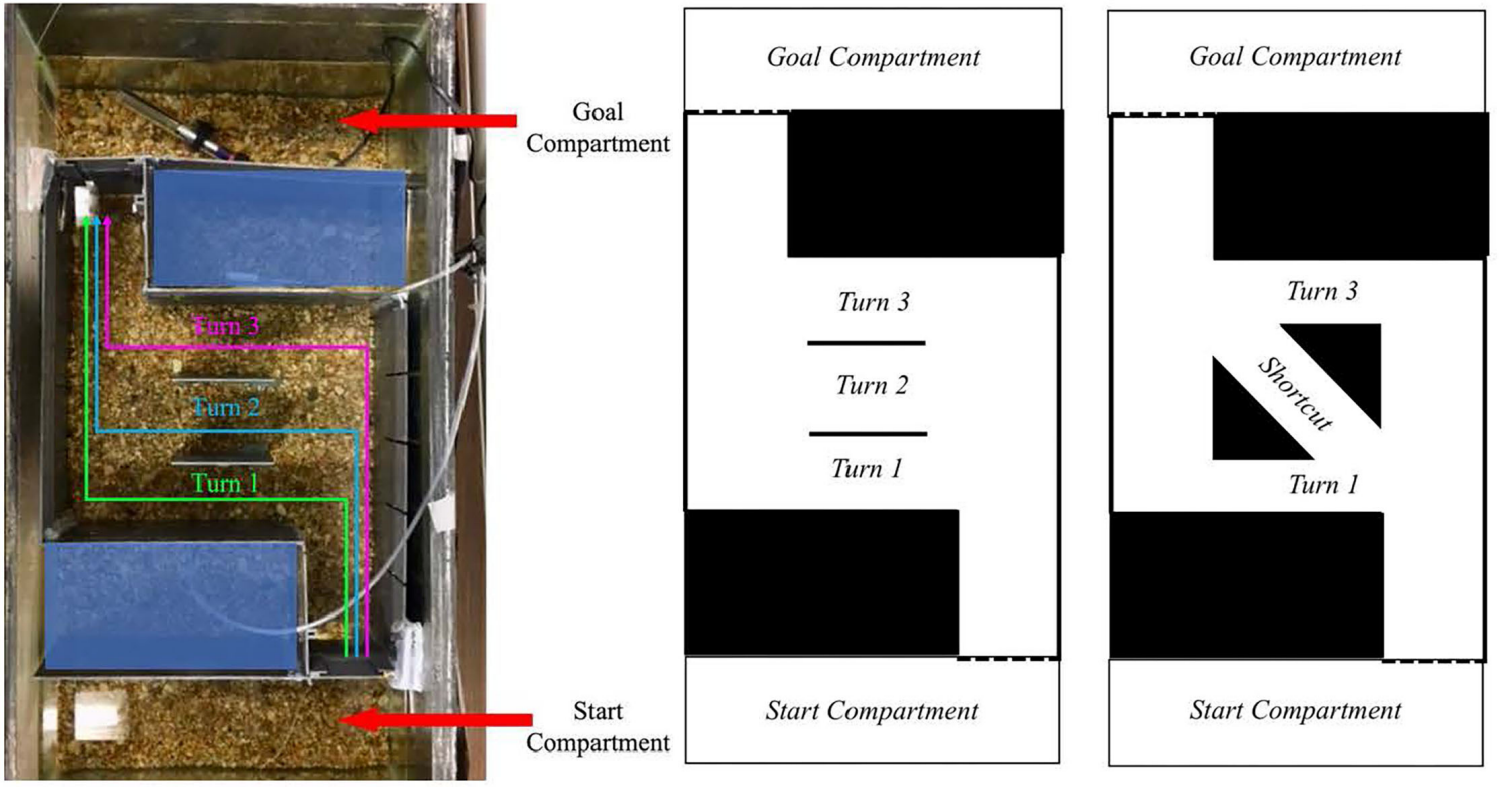
**(Left)** Overhead view of the fish tank during a test trial. There were three possible turn options, all of equivalent length. During familiarization, the middle two walls, which create the turns, were not present. **(Right)** Schematic of the maze used and the proposed maze with a shortcut. Photo: Victoria Braithwaite; diagrams: Natalie Schwob.

While not everyone agrees on what a heuristic exactly is [see (64, 65)], heuristics are often studied in the context of reducing the cognitive burden of decision-making to arrive at an adequate solution. However, when choices are devoid of consequences, humans still conform to a consistent solution to a problem. Christenfeld (66) explored human choices from identical options by presenting participants with a maze containing three path options that yielded equivalent solutions with respect to distance traveled. Despite the apparent equivalence between options, participants preferred to take the final turn rather than the two turns that were available earlier (see Figure 1 for abstraction of maze). This heuristic has been replicated and termed action continuation (67). Rather than changing routes between multiple iterations of the maze, participants tend to use the same strategy over multiple instances (67), perhaps as a means of reducing the cognitive burdens associated with generating a new plan by reusing a previously executed plan (68, 69). Humans undergo an automatic process of decision-making, despite no consequence of any decision (66, 67). However, heuristics can also lead to suboptimal strategies as well, for example, the peak-end effect discussed above (22).

Heuristics are widespread across different species of animals. Humans and capuchins (*Sapajus apella*) are suspectable to framing effects. Humans are more likely to take the same gamble when it is described as a likelihood of winning vs. a likelihood of losing (70). Similarly, capuchins have a preference receiving food framed as a gain (seeing one piece and receiving two 50% of the time) vs. a loss (seeing two pieces and receiving two 50% of the time) despite earning the same amount of food regardless of framing (71). Additionally, when presented with an undesirable third option, humans (72), honeybees (*Apis mellifera*) and gray jays (*Perisoreus canadensis*) violate the principle of irrelevant alternatives which states that a preference between two options should not change depending on the presence or absence of an additional option (73). Thus, there is evidence to suggest that some heuristics are evolutionarily conserved across multiple species and even taxa.

Our proposed study of flexibility utilizes the action continuation heuristic in a maze navigation task with fish (see Figure 1). This task would answer two questions: Do fish, like humans, use a heuristic to solve a navigation task in which all choices incur an equivalent cost? And if their choices conform to a heuristic, can fish change their behavior when presented with a more efficient shortcut? This type of flexibility would demonstrate an awareness that the context has changed with the availability of a more efficient option. To understand this process, we collected preliminary data on 12 zebra fish (*Danio rerio*) using a maze derived from experiments with humans (66, 67). The maze consisted of an entrance compartment that opened to a long straight arm, containing three possible turns into a shorter arm. From there, a longer arm led to the exit into a goal compartment where the fish received food (see Figure 1). After each fish ran the maze twice, we found that the fish preferred to take the first or last turn (50 and 41.66% respectively). Here, we were able to determine that the fish show a first turn and last turn bias, and mostly ignored the middle turn (8.33%). If a better, more efficient option were to become available, ignoring their original preference for this better solution would show flexibility. The original path would become suboptimal, though still a possible solution to the maze. To test whether fish would alter their behavior in this way, we planned to add a shortcut to the maze in place of the unpreferred middle turn. If the fish are not flexible in their decision making, they would continue to utilize their heuristic and take the same turn as the previous maze, not taking advantage of the shortcut option. If the fish were able to notice this change in the maze and take the shortcut, it would show they have a representation of where the end of the maze is in relation to their current location, and that they recognize the shortcut would be a faster route than the other two equivalent paths. However, if the fish fail to take the shortcut, this would not necessarily indicate that they lack flexibility.

Thus, we had a third condition planned to provide the fish with additional maze information. Rather than giving them experience in the tank with a shortcut, the fish would be placed in a tank with an overhead view that would allow visual access to the maze prior to entering it. We were interested in seeing if the fish would map their route prior to entering the maze for the first time by updating their representation of the maze where all options are no longer equivalent. Chimpanzees (*Pan troglodytes*), who most would agree are conscious and flexible, successfully transform spatial information. After viewing a hidden location of a juice bottle on a scaled-down model of their yard, chimpanzees directly went to the corresponding location outside (74). Rather than translating information from a model to the actual location, the fish in our study would simply need to update their representation of the maze. If the fish were to behave flexibly, they would prioritize taking the shortcut over taking the previously successful path. As noted above, goby fish demonstrate this sort of flexible behavior as part of their repertoire. If other fish species can also learn to map a maze by swimming above it, this is further evidence of flexibility over variability in behavior^1^.

While we think a navigation task can add valuable information regarding flexibility and consciousness, we want to stress that not a single test is adequate for all species. Instead, we are suggesting flexibility should be built into the search of indicators of potential consciousness in animals. For example, the ecology of the species needs to be considered. Research from Braithwaite and Girvan (75) found that three-spined sticklebacks (*Gasterosteus aculeatus*) can learn to use flow direction as a navigational tool to locate a hidden food patch. When the flow direction was reversed, fish reared in a river environment where water is often flowing were able to adapt to the reversed contingency faster than fish reared in a static, pond environment. Using water flow as a spatial cue could be appropriate in river fish, though it may not be for a species who does not live in a similar environment. To demonstrate flexibility and consciousness, multiple species-appropriate tests would be required: a mass of evidence is essential.

## Flexibility and Consciousness

This final section has the difficult task of explaining why flexibility indicates consciousness. The ability to evaluate complex situations and shift goals and actions accordingly is certainly adaptive. But why think it is conscious? As noted above, this is the explanatory gap. We need to explain why subjective experience is necessary for flexibility. While a full argument is beyond the scope of this article, the essential move is a description of consciousness in terms of function. On the Temporal Representation Theory, consciousness represents the present moment in order to facilitate flexible action (13). The function of consciousness is rooted in the adaptive benefit of flexibility.

In order to assess a situation and adapt goals and actions appropriately, an animal needs to represent how things are now. Task-relevant stimuli are selected and integrated into representations of the animal's external and internal environment to provide an ongoing update of current conditions. Because stimulus processing and coordination take time, conscious representation generally lags somewhat from the timing of the original signal. In time-sensitive contexts such as motion-detection, however, predictive processes anticipate shifts in order to better represent the stimulus location (11, 76–78). Consciousness is the integration of top-down and bottom-up stimulus selection into the best representation of the world at the present moment.

There are two main reasons that a representation of the world at the present moment is necessary for flexible action. The conceptual reason is that a division between past and future is needed to open the possibility of alternative actions. Simple associative response follows algorithmically from stimulus input. Past training determines behavior in a 1–1 input-output relation. In contrast, flexible behavior involves a more complex and dynamic mapping relation from input to output. Past training figures prominently, of course, in weighting various goals and their motivational valence. The critical difference is how the past is utilized: in simple conditioning, the past determines a single response, whereas in flexible response, past learning is one factor in assessing the situation. The distinction between past and future is probably not explicit for non-linguistic creatures (79); only an ability to represent now relative to not now is involved in consciousness, as described earlier in the representation of absent objects.

The second, pragmatic reason for regularly updating a representation of the world is that decision-making processes need information about what is happening now in order to ensure that progress is continuing on task or to initiate a change in course. Accumulating evidence suggests that decisions are made unconsciously, and action is initiated prior to the conscious feeling of decision (80–82). Nonetheless, conscious monitoring of the current situation coordinates information about obstacles and opportunities relative to ongoing goal pursuit. Consciousness grounds decision-making in the present by means of a unified representation of relevant information [see (83) for a similar view].

Of course, representations of the past and the future can also be conscious, so it may seem that consciousness cannot be adequately defined as a representation of the present moment. However, memory and imagination are forms of self-consciousness that arguably depend on the prior development of a representation of presence (84). Moreover, there is reason to think that these explicit representations of past and future are embedded in a representation of the present moment. That is, a conscious memory of last summer's vacation is a matter of representing this past event as in some way present (85). As in the case of dual systems, evidence for a sophisticated mental ability such as self-consciousness is sufficient but not necessary for the attribution of consciousness.

Throughout this article we have argued for a characterization of consciousness that falls between an anthropocentric approach like self-reflection and an anthropomorphic approach like basic association. In the previous two sections we highlighted the functional connections between flexible behavior and consciousness. It may be tempting to stop there and not attempt to situate flexibility within a particular theory of consciousness. Although great advances have been made in the science of consciousness (86), controversy and confusion continue to plague the field, particularly regarding animal consciousness.

At this point it is appropriate to credit Victoria for inspiring us with the courage to take this difficult and important additional step. Victoria's commitment to both science and animal welfare convinced her that the question of whether fish consciously feel pain should be answered, and she set about to collect the people and data to help her find the answer. The final answer will require further research to develop tests applicable to animals of widely varying groups. No single test is likely to be decisive. Instead, we should expect a gradual convergence of evidence—behavioral, physiological, and evolutionary—to develop in favor of or against ascription of consciousness in any particular case. We have suggested four general ways to test for flexibility. Physiological evidence for systems that enable flexibility—motivation and attention systems, for example—would also add strength to a case for ascribing consciousness. Evolutionary considerations may provide the most compelling means of determining where consciousness is found in the animal kingdom. According to the Temporal Representation Theory, consciousness is a representation of the present moment, and the capacity for this form of representation evolved to facilitate flexible action. If this view is correct, demonstration of flexible behavioral responses by fish or other animals is evidence of consciousness.

## Data Availability Statement

The original contributions presented in the study are included in the article/supplementary material, further inquiries can be directed to the corresponding author.

## Author Contributions

PD wrote most of the text. NS and DW each wrote one section. DW provided comments and edits. NS coined the term fishnition. The ideas were a collaboration of all authors.

## Conflict of Interest

The authors declare that the research was conducted in the absence of any commercial or financial relationships that could be construed as a potential conflict of interest.

## Publisher's Note

All claims expressed in this article are solely those of the authors and do not necessarily represent those of their affiliated organizations, or those of the publisher, the editors and the reviewers. Any product that may be evaluated in this article, or claim that may be made by its manufacturer, is not guaranteed or endorsed by the publisher.

## Footnotes

^1^Sadly, we were not able to complete the shortcut experiment nor the overhead view experiment. Victoria conducted the initial navigation task but was unable to set up and run the other conditions. We encourage other investigators to take up this research and would happily share our plans for these experiments.

## References

[1] Birch J. The search for invertebrate consciousness. Noûs. (2020) 1–21. 10.1111/nous.12351 PMC7612530 35321054

[2] Shevlin H. Non-human consciousness and the specificity problem: a modest theoretical proposal. Mind Lang. (2021) 36:297–314. 10.1111/mila.12338

[3] Nagel T. (1974). What is it like to be a bat? Philos Rev. 83:435. 10.2307/2183914

[4] Block N. On a confusion about a function of consciousness. Behav Brain Sci. (1995) 18:227–47. 10.1017/S0140525X0003818830886898

[5] Feinberg TE Mallatt J. Phenomenal consciousness and emergence: eliminating the explanatory gap. Front Psychol. (2020) 11:1041. 10.3389/fpsyg.2020.01041 32595555 PMC7304239

[6] Evans JSBT Over DE. Rationality and Reasoning. Mahwah, NJ: Psychology/Erlbaum (1996).

[7] Tversky A Kahneman D. Judgment under uncertainty: heuristics and biases. Science. (1974) 185:1124–31. 10.1126/science.185.4157.1124 17835457

[8] Clark RE Squire LR. Classical conditioning and brain systems: the role of awareness. Science. (1998) 280:77–81. 10.1126/science.280.5360.77 9525860

[9] Droege P Weiss DJ Schwob N Braithwaite V. Trace conditioning as a test for animal consciousness: a new approach. Anim Cogn. (2021) 2021:1–6. 10.1007/s10071-021-01522-3 33983542

[10] Droege P. Caging the Beast: A Theory of Sensory Consciousness. Amsterdam: John Benjamins Publishing (2003).

[11] Droege P. Now or never: how consciousness represents time. Conscious Cogn. (2009) 18:78–90. 10.1016/j.concog.2008.10.006 19041266

[12] Droege P Braithwaite VA. A framework for investigating animal consciousness. In: Lee G Illes J Ohl F editors. Ethical Issues in Behavioral Neuroscience. Berlin Heidelberg: Springer (2014). p. 79–98 10.1007/7854_2014_278 24510302

[13] Droege P. Evolution of Consciousness: Representing the Present Moment. New York: Bloomsbury Academic (2021).

[14] Braithwaite VA. Do Fish Feel Pain? Oxford: Oxford University Press (2010).

[15] Braithwaite VA Huntingford F van den Bos R. Variation in emotion and cognition among fishes. J Agric Environ Ethics. (2013) 26:7–23. 10.1007/s10806-011-9355-x

[16] Low P Panksepp J Reiss D Edelman D Van Swinderen B Koch C. Cambridge Declaration on Consciousness in Non-Human Animals. In: Francis Crick Memorial Conference, Cambridge (2012). Available online at: http://fcmconference.org/ (accessed July 2, 2021)

[17] Graziano MSA Guterstam A Bio BJ Wilterson AI. Toward a standard model of consciousness: reconciling the attention schema, global workspace, higher-order thought, and illusionist theories. Cogn Neuropsychol. (2020) 37:155–172. 10.1080/02643294.2019.1670630 31556341

[18] Evans JSBT. In two minds: dual-process accounts of reasoning. Trends Cogn Sci. (2003) 7:454–9. 10.1016/j.tics.2003.08.012 14550493

[19] Stanovich KE. Who is Rational?: Studies of Individual Differences in Reasoning. Mahwah, NJ: Lawrence Erlbaum Associates Publishers (1999).

[20] Kahneman D. Thinking, Fast and Slow. New York, NY: Farrar, Straus and Giroux (2013).

[21] Osman M. An evaluation of dual-process theories of reasoning. Psychonom Bull Rev. (2004) 11:988–1010. 10.3758/BF03196730 15875969

[22] Kahneman D Fredrickson BL Schreiber CA Redelmeier DA. When more pain is preferred to less: adding a better end. Psychol Sci. (1993) 4:401–5. 10.1111/j.1467-9280.1993.tb00589.x

[23] Kahneman D Thaler RH. Anomalies: utility maximization and experienced utility. J Econ Perspect. (2006) 20:221–34. 10.1257/089533006776526076

[24] Xu ER Knight EJ Kralik JD. Rhesus monkeys lack a consistent peak-end effect. Q J Exp Psychol. (2011) 64:2301–15. 10.1080/17470218.2011.591936 21929474 PMC3437997

[25] Blanchard TC Wolfe LS Vlaev I Winston JS Hayden BY. Biases in preferences for sequences of outcomes in monkeys. Cognition. (2014) 130:289–99. 10.1016/j.cognition.2013.11.012 24374208 PMC3969290

[26] Egan Brad LC Lakshminarayanan VR Jordan MR Phillips WC Santos LR. The evolution and development of peak–end effects for past and prospective experiences. J Neurosci Psychol Econ. (2016) 9:1. 10.1037/npe0000048

[27] Frederick S Loewenstein G. Conflicting motives in evaluations of sequences. J Risk Uncertain. (2008) 37:221–35. 10.1007/s11166-008-9051-z

[28] Santos LR Rosati AG. The evolutionary roots of human decision making. Annu Rev Psychol. (2015) 66:321–47. 10.1146/annurev-psych-010814-015310 25559115 PMC4451179

[29] Heyes C. Simple minds: a qualified defence of associative learning. Philos Trans R Soc B: Biol Sci. (2012) 367:2695–703. 10.1098/rstb.2012.0217 22927568 PMC3427553

[30] Clayton NS Dickinson A. Episodic-like memory during cache recovery by scrub jays. Nature. (1998) 395:272–4. 10.1038/26216 9751053

[31] Roberts WA. Are animals stuck in time? Psychol Bull. (2002) 128:473–89. 10.1037/0033-2909.128.3.473 12002698

[32] Droege P. Assessing evidence for animal consciousness: the question of episodic memory. In: Smith JA Mitchell RW editors. Experiencing Animal Minds: An Anthology of Animal-Human Encounters. Columbia: Columbia University Press (2012), p. 231–245.

[33] Griffin DR Speck GB. New evidence of animal consciousness. Anim Cogn. (2004) 7:5–18. 10.1007/s10071-003-0203-x 14658059

[34] Dylla KV Galili DS Szyszka P Lüdke A. Trace conditioning in insects—keep the trace! Front Physiol. (2013) 4:67. 10.3389/fphys.2013.00067 23986710 PMC3750952

[35] Szyszka P Demmler C Oemisch M Sommer L Biergans S Birnbach B. Mind the gap: olfactory trace conditioning in honeybees. J Neurosci. (2011) 31:7229–39. 10.1523/JNEUROSCI.6668-10.2011 21593307 PMC6622586

[36] Bekinschtein TA Peeters M Shalom D Sigman M. Sea slugs, subliminal pictures, and vegetative state patients: boundaries of consciousness in classical conditioning. Front Psychol. (2011) 2:337. 10.3389/fpsyg.2011.00337 22164148 PMC3230906

[37] Lovibond PF Shanks DR. The role of awareness in Pavlovian conditioning: empirical evidence and theoretical implications. J Exp Psychol Anim Behav Process. (2002) 28:3–26. 10.1037/0097-7403.28.1.3 11868231

[38] Rose JD Arlinghaus R Cooke SJ Diggles BK Sawynok W Stevens ED. Can fish really feel pain? Fish Fish. (2014) 15:97–133. 10.1111/faf.12010

[39] Treisman A. The perception of features and objects. In: Baddeley A Weiskrantz L editors. Attention: Selection, Awareness and Control. Oxford: Clarendon Press (1993), p. 5–35.

[40] van Boxtel JJA Tsuchiya N Koch C. Consciousness and attention: on sufficiency and necessity. Front Psychol. (2010) 1:217. 10.3389/fpsyg.2010.00217 21833272 PMC3153822

[41] Jennings CD. Consciousness without attention. J Am Philos Assoc. (2015) 1:276–95. 10.1017/apa.2014.1430886898

[42] Koch C Tsuchiya N. Attention and consciousness: two distinct brain processes. Trends Cogn Sci. (2007) 11:16–22. 10.1016/j.tics.2006.10.012 17129748

[43] Christian KM Thompson RF. Neural substrates of eyeblink conditioning: acquisition and retention. Learn Memory. (2003) 10:427–55. 10.1101/lm.59603 14657256

[44] Tononi G Koch C. Consciousness: Here, There But Not Everywhere. (2014). ArXiv:1405.7089 [q-Bio]. http://arxiv.org/abs/1405.7089

[45] Duran E Ocana FM Broglio C Rodriguez F Salas C. Lateral but not medial telencephalic pallium ablation impairs the use of goldfish spatial allocentric strategies in a “‘hole-board”' task. Behav Brain Res. (2010) 214:480–7. 10.1016/j.bbr.2010.06.010 20600353

[46] Rodriguez F Duran E Vargas JP Torres B Salas C. Performance of goldfish trained in allocentric and egocentric maze procedures suggests the presence of a cognitive mapping system in fishes. Anim Learn Behav. (1994) 22:409–20. 10.3758/BF03209160

[47] Bshary R Grutter AS. Punishment and partner switching cause cooperative behavior in a cleaning mutualism. Biol Lett. (2005) 1:396–99. 10.1098/rsbl.2005.0344 17148216 PMC1626376

[48] Bshary R Würth M. Cleaner fish *Labroides dimidiatus*. manipulate client reef fish by providing tactile stimulation. Proc R Soc Lond B. (2001) 268:1495–501. 10.1098/rspb.2001.1495 11454294 PMC1088769

[49] Tebbich S Bshary R Grutter AS. Cleaner fish *Labroides dimidiatus*. recognise familiar clients. Anim Cogn. (2002) 5:139–45. 10.1007/s10071-002-0141-z 12357286

[50] Grosenick L Clement TS Fernald RD. Fish can infer social rank by observation alone. Nature. (2007) 445:419–32. 10.1038/nature05646 17251980

[51] Pasko Ł. Tool-like behavior in the sixbar wrasse, *Thalassoma hardwicke*. (Bennett, 1830). Zoo Biol. (2010) 29:767–73. 10.3389/10.1002/zoo.20307 20095003

[52] Brown C. Tool use in fishes. Fish Fish. (2012) 13:105–15. 10.1111/j.1467-2929.2011.00451.x

[53] Dickinson A Balleine B. Motivational control of goal-directed action. Anim Learn Behav. (1994) 22:1–18. 10.3758/BF03199951

[54] Aronson LR. Orientation and jumping behavior in the Gobiid fish, *Bathygobius soporator*. Am Museum Novit. (1951) 1486:1–22. 5288865 10.1111/j.1749-6632.1971.tb13110.x

[55] Aronson LR. Further studies on orientation and jumping behavior in the Gobiid fish, *Bathygobius soporator*. Ann N Y Acad Sci. (1971) 188, 378–392. 10.3389/10.1111/j.1749-6632.1971.tb13110.x 5288865

[56] Braithwaite V De Perera TB. Short-range orientation in fish: how fish map space. Mar Freshw Behav Physiol. (2006) 39:37–47. 10.1080/10236240600562844

[57] Ginsburg S Jablonka E. The Evolution of the Sensitive Soul: Learning and the Origins of Consciousness. New York: MIT Press (2019).

[58] Birch J Ginsburg S Jablonka E. Unlimited associative learning and the origins of consciousness: a primer and some predictions. Biol Philos. (2020) 35:56. 10.1007/s10539-020-09772-0 33597791 PMC7116763

[59] Droege P. The lives of others: pain in non-human animals. In: Corns J editor. The Routledge Handbook of Philosophy of Pain. Taylor and Francis (2017), p. 191–99.

[60] Locked-in syndrome,. NIH. (2021). Available online at: https://rarediseases.info.nih.gov/diseases/6919/locked-in-syndrome (accessed February 1, 2021)

[61] Olton DS Collison C Werz MA. Spatial memory and radial arm maze performance of rats. Learn Motiv. (1977) 8:289–314. 10.1016/0023-9690(77)90054-624454487

[62] Tolman EC Ritchie BF Kalish D. Studies in spatial learning. I Orientation and the short-cut. J Exp Psychol. (1946) 36:13–24. 10.1037/h0053944 21015338

[63] Bett NN Hinch SG. Olfactory navigation during spawning migrations: A review and introduction of the Hierarchical Navigation Hypothesis. Biol Rev Camb Philos Soc. (2016) 91:728–59. 10.1111/brv.12191 25923999

[64] Gigerenzer G Todd PM. Fast and frugal heuristics: the adaptive toolbox. In: Gigerenzer G Todd PM editors. Simple Heuristics That Make us Smart. Oxford: Oxford University Press (1999), p. 3–34.

[65] Marsh B. Do animals use heuristics? J Bioecon. (2002) 4:49–56. 10.1023/A:1020655022163

[66] Christenfeld N. Choices from identical options. Psychol Sci. (1995) 6:50–5. 10.1111/j.1467-9280.1995.tb00304.x

[67] van Tilburg WA Igou ER. Moving onwards: an action continuation strategy in finding the way. J Behav Decis Mak. (2014) 27:408–18. 10.1002/bdm.181725855820

[68] Valyear KF Fitzpatrick AM Dundon NM. Now and then: hand choice is influenced by recent action history. Psychonom Bull Rev. (2018) 26:305–14. 10.3758/s13423-018-1510-1 30039397 PMC6424939

[69] Weiss DJ Wark J. Hysteresis effects in a motor task with cotton-top tamarins (*Sanguinus oedipus*). J Exp Psychol Anim Behav Process. (2009) 35:427–33. 10.1037/a0013964 19594287

[70] Levin IP Schneider SL Gaeth GJ. All Frames are not created equal: a typology and critical analysis of framing effects. Organ Behav Hum Decis Process. (1998) 76:149–88. 10.1006/obhd.1998.2804 9831520

[71] Chen MK Lakshminarayanan V Santos LR. How basic are behavioral biases? Evidence from capuchin monkey trading behavior. J Politic Econ. (2006) 114:517–37. 10.1086/503550

[72] Huber J Payne JW Puto C. Adding asymmetrically dominated alternatives: violations of regularity and the similarity hypothesis. J Consum Res. (1982) 9:90–8. 10.1086/208899

[73] Shafir S Waite TA Smith BH. Context-dependent violations of rational choice in honeybees (*Apis mellifera*) and gray jays (*Perisoreus canadensis*). Behav Ecol Sociobiol. (2002) 51:180–7. 10.1007/s00265-001-0420-8

[74] Kuhlmeier VA Boysen ST. Chimpanzees (*Pan troglodytes*) recognize spatial and object correspondences between a scale model and its referent. Psychol Sci. (2002) 13:60–3. 10.1111/1467-9280.00410 11892779

[75] Braithwaite VA Girvan JR. Use of water flow direction to provide spatial information in a small-scale orientation task. J Fish Biol. (2003) 63:74–83. 10.1111/j.1095-8649.2003.00218.x

[76] Grush R. On the temporal character of temporal experience, its scale non-invariance, and its small scale structure. Manuscript. (2016). 10.21224/P4WC73

[77] Heusden E van Harris AM Garrido MI Hogendoorn H. Predictive coding of visual motion in both monocular and binocular human visual processing. J Vis. (2019) 19, 3–3. 10.1167/19.1.3 30630191

[78] Parsons BD Novich SD Eagleman DM. Motor-sensory recalibration modulates perceived simultaneity of cross-modal events at different distances. Percept Sci. (2013) 4:46. 10.3389/fpsyg.2013.00046 23549660 PMC3582016

[79] Suddendorf T. The Gap: The Science of What Separates Us From Other Animals. New York, NY: Basic Books (2013).

[80] Haggard P. Human volition: towards a neuroscience of will. Nat Rev Neurosci. (2008) 9:934–46. 10.1038/nrn2497 19020512

[81] Swinburne R (editor). Free Will and Modern Science. Oxford: Oxford University Press (2012).

[82] Wegner DM. The Illusion of Conscious Will. Cambridge: MIT Press (2003).

[83] Pennartz CMA Farisco M Evers K. Indicators and criteria of consciousness in animals and intelligent machines: an inside-out approach. Front Syst Neurosci. (2019) 13:25. 10.3389/fnsys.2019.00025 31379521 PMC6660257

[84] Droege P. Memory and consciousness. Philos Sci. (2013) 17:171–93. 10.4000/philosophiascientiae.865

[85] Droege P. Consciousness and memory. In: Bernecker S Michaelian K editors. The Routledge Handbook of Philosophy of Memory. Boca Raton: Routledge, Taylor and Francis Group (2017), p. 103–112.

[86] Overgaard M. The status and future of consciousness research. Front Psychol. (2017) 8:1719. 10.3389/fpsyg.2017.01719 29066988 PMC5641373

